# The UPBEAT Nurse-Delivered Personalized Care Intervention for People with Coronary Heart Disease Who Report Current Chest Pain and Depression: A Randomised Controlled Pilot Study

**DOI:** 10.1371/journal.pone.0098704

**Published:** 2014-06-05

**Authors:** Elizabeth A. Barley, Paul Walters, Mark Haddad, Rachel Phillips, Evanthia Achilla, Paul McCrone, Harm Van Marwijk, Anthony Mann, Andre Tylee

**Affiliations:** 1 Florence Nightingale School of Nursing and Midwifery, James Clerk Maxwell Building, King's College London, London, United Kingdom; 2 Dorset HealthCare University NHS Foundation Trust, Weymouth and Portland Community Mental Health Team, Mental Health Centre, Weymouth, Dorset, United Kingdom; 3 School of Health Sciences, City University London, London, United Kingdom; 4 Health Services and Population Research Department, Institute of Psychiatry, King's College London, London, United Kingdom; 5 Department of General Practice & Elderly Care Medicine and the EMGO Institute for Health and Care Research of VU University Medical Centre, Amsterdam, The Netherlands; Universidad de Valladolid, Spain

## Abstract

**Background:**

Depression is common in people with coronary heart disease (CHD) and associated with worse outcome. This study explored the acceptability and feasibility of procedures for a trial and for an intervention, including its potential costs, to inform a definitive randomized controlled trial (RCT) of a nurse-led personalised care intervention for primary care CHD patients with current chest pain and probable depression.

**Methods:**

Multi-centre, outcome assessor-blinded, randomized parallel group study. CHD patients reporting chest pain and scoring 8 or more on the HADS were randomized to personalized care (PC) or treatment as usual (TAU) for 6 months and followed for 1 year. Primary outcome was acceptability and feasibility of procedures; secondary outcomes included mood, chest pain, functional status, well being and psychological process variables.

**Result:**

1001 people from 17 General Practice CHD registers in South London consented to be contacted; out of 126 who were potentially eligible, 81 (35% female, mean age = 65 SD11 years) were randomized. PC participants (n = 41) identified wide ranging problems to work on with nurse-case managers. Good acceptability and feasibility was indicated by low attrition (9%), high engagement and minimal nurse time used (mean/SD = 78/19 mins assessment, 125/91 mins telephone follow up). Both groups improved on all outcomes. The largest between group difference was in the proportion no longer reporting chest pain (PC 37% *vs* TAU 18%; mixed effects model OR 2.21 95% CI 0.69, 7.03). Some evidence was seen that self efficacy (mean scale increase of 2.5 *vs* 0.9) and illness perceptions (mean scale increase of 7.8 *vs* 2.5) had improved in PC *vs* TAU participants at 1 year. PC appeared to be more cost effective up to a QALY threshold of approximately £3,000.

**Conclusions:**

Trial and intervention procedures appeared to be feasible and acceptable. PC allowed patients to work on unaddressed problems and appears cheaper than TAU.

**Trial Registration:**

Controlled-Trials.com ISRCTN21615909

## Introduction

The point prevalence of depression in coronary heart disease (CHD) patients has been estimated at 20% in patients with acute myocardial infarction[1] and 9% in a community sample of people with chronic disease[2]. Depression increases the incidence of coronary symptoms and death in CHD patients[3]. Established depression treatments (antidepressants and psychotherapies) have only a moderate effect on improving mood and reducing cardiac events but no effect on mortality[4]. More complex systems approaches, such as collaborative care, may be more successful[5]. Collaborative care involves structured management plans, scheduled follow-ups and enhanced inter-professional working, i.e. case manager/practice nurse, primary care practitioner and mental health specialist working together [6], and has been shown to improve indirect cardiac outcomes such as disease control and 10-year cardiovascular disease risk as well as depression[6], [7] in people with CHD and/or diabetes.

The above trials were conducted in the USA[6] and Australia[7]where healthcare systems differ from the NHS, in particular, there is less interprofessional working[8]. The UPBEAT-UK programme of research[9] was funded by the National Institute for Health Research (NIHR) to explore the relationship between CHD and depression and to develop a new intervention feasible for use in UK primary care.

The intervention examined in this pilot study was developed in line with accepted guidance that interventions for health improvement should be informed by evidence and based on a theory of how they cause change “so that weak links in the causal chain can be identified and strengthened” [10]. Using guidelines for developing and evaluating complex interventions[11], we conducted and published a systematic review[12] and qualitative research with patients[13] and primary care staff[8] to develop a novel intervention. We used iterative literature review to synthesise our empirical work and to identify evidence and theory to inform the intervention, which was modified following a patient focus group; this process has been reported[11].

In summary, our development studies were consistent in indicating that there is wide variation in the problems that depressed people with CHD report as contributing to their low mood, but that social problems are very common and that primary care staff are unsure how to manage them. Another key finding was that GPs reported having no time to deliver depression interventions; this was similarly the case for practice nurses, who also reported a lack of confidence in depression management[8], [11], [12]. It was clear from these findings that the UPBEAT intervention needed to be tailored to individual need, brief to deliver and compatible with current practice.

An established component of chronic disease management in primary care is the provision of self management support; this means enabling patients to take better care of themselves, for instance by providing information and helping them to change unhealthy behaviours. Our literature review showed that accepted health psychology models agree on two factors important for behaviour change: belief in the importance of an outcome and belief in capacity to succeed (self efficacy)[11]; we therefore incorporated established behaviour change techniques, such as goal setting and action planning, which aim to enhance the patient's self efficacy to achieve their desired outcomes.

In line with our aim to develop an intervention feasible for UK primary care, our intervention is designed to enhance usual care. Practice nurses are already responsible for supporting CHD patients' self management and have been found to be effective case managers in the treatment of chronic depression[14]. In our intervention, the practice nurse helps the patient to identify problems important to them and then draws on a ‘tool box’ of techniques to help the patient address these problems. The nurse also sign posts the patient to relevant existing sources of help and makes referrals to other agencies where appropriate. Hence the practice nurses, acting as case managers, deliver care that is more holistic than that currently provided.

Our findings of uncertainty around the needs of depressed CHD patients and lack of confidence in managing them led us to conduct a pilot study, in which the intervention was delivered by nurse researchers, that would inform the best methods for a definitive RCT[9]. We knew, from our cross-sectional analyses of the UPBEAT cohort study [15], that CHD patients have high levels of co-morbidity and that for some patients on GP CHD registers any cardiac event would have been several years previously; we therefore only recruited those patients with symptomatic CHD (i.e. reporting current chest pain) in order to ensure that they would understand the intervention in terms of their heart disease. The current study therefore explored the acceptability and feasibility of procedures for a trial and for the intervention, including its potential costs, to inform a definitive RCT of a novel, practice nurse-led personalised care intervention for CHD patients who have at least probable depression and current chest pain.

To this end, we examined: a) the rate of participant recruitment and reasons for non-participation; b) research procedures including randomization, blinding and data collection; c) the acceptability and feasibility of the intervention; d) potential costs of the intervention; and e) the appropriateness of primary outcome and secondary outcome measures in relation to patients' reported problems. Mediator analyses were conducted to identify trends between the groups in changes in self efficacy and illness beliefs and the impact of these on depression outcomes and to determine whether therapist effects or intervention intensity (nurse time) are likely to be important.

## Methods

The protocol for this trial and supporting CONSORT checklist are available as supporting information; see Checklist S1 and Protocol S1.

### Ethics Statement

The study was reviewed and approved by the South East London Research Ethics Committee (REC Ref no: 10/H0808/5). Written informed consent was obtained from all study participants.

### Pilot study design

This was a multi-centre, outcome assessor blinded, parallel group study with randomisation at the patient level with 1∶1 allocation. The study protocol has been published [16] and the pilot trial is registered with Current Controlled Trials: ISRCTN 21615909.

### Deviations from original protocol

The original protocol is available as online supporting information (Protocol S1). In this and the published protocol [15], one aim was to explore potential primary outcomes, we focused on depression as measured by the Hospital Anxiety and Depression scale (HADS). In this report, since we also had collected another measure of depression - the PHQ-9 (as per protocol), we also explored this as a primary outcome as a further examination of the appropriateness of the HADS as a primary outcome for a future trial. In addition, during the pilot study the intervention group participants identified pain as an important problem contributing to their depression; in this report, we therefore also examined our measure of chest pain as a potential primary outcome. In our original protocol, we referred to our intervention as ‘case management’, however following our intervention development process, summarized here and reported in detail elsewhere [10], the term ‘personalised care’ was considered more appropriate. Finally, our original protocol does not detail the specific analysis plan reported here. All data will be deposited in an accessible archive at King's College London.

### Study setting

Practices in South London were recruited via the Greater London Primary Care Research Network (PCRN-GL). To be included, the practice had to keep a register of patients with CHD for the Quality and Outcomes Framework (QOF)[17] and be willing to liaise over patients in the PC arm when necessary.

### Participants

Adults with symptomatic CHD (registered on GP CHD QOF register and reporting chest pain), reporting depression symptoms were eligible. All patients on practice case registers for CHD were asked by their GP for consent to contact from a researcher. Those consenting were contacted by a researcher and assessed for depressive symptoms using the PHQ-2[18] and for symptoms of current chest pain using the Modified Rose Angina Questionnaire[19]. Patients scoring 3 or more on the PHQ-2 (i.e. a score of 3 is considered the optimal cut point for depression screening and which indicates further screening is necessary), and who reported current chest pain were assessed using the Hospital Anxiety and Depression Scale (HADS)[20]. If they scored ≥8 on the depression scale of HADS (HADS-D)(i.e. the score validated to indicate probable depression caseness) they were eligible to participate. Patients who were temporary registrants, actively suicidal, suffering from psychotic depression, non-English speaking or currently hospitalised were excluded. Other physical and mental co-morbidities were allowed. Participants were recruited between October 2010 and June 2011.

### Intervention: Personalised care (PC)

Full details have been reported[11]. In brief, the nurse acting as case manager conducts a standardised, face to face, bio-psychosocial assessment (including physical and mental health, difficulties with current treatment regimens, problems with daily activities and social problems). Patients are then helped to identify up to three problems which they consider contribute to their depression and which they most want to address. The nurse-case managers provide information, sign-post patients to existing resources (e.g. leisure centres, social clubs, Improving Access to Psychological Therapy (IAPT) services) and use evidence based behaviour change techniques to help patients set and achieve goals. The underlying intention of the intervention is to increase the patient's self-efficacy to achieve their desired goals (as opposed to goals determined by others such as symptom management or reduction of cardiac risk factors).

Details of the assessment and action plan are recorded in a ‘personalised health plan’ which the patient holds. Follow up interviews to determine progress and/or set new goals are conducted via telephone. Calls are planned to last 15 minutes and are scheduled weekly initially then at increasing intervals according to patient need. During the 6 month intervention period weekly meetings were held with research team clinicians (a GP academic (AT) and two psychiatrists (AM, PW) to ensure fidelity to the intervention.

### Control: treatment as usual (TAU)

All patients received primary care TAU from their GP and/or Practice Nurse (PN); this may include specific depression intervention such as antidepressant prescription or referral to talking therapy.

### Measurement

Data were collected face to face at baseline and via telephone at 1, 6 and 12 months post randomisation. Data collection was completed by July 2012.

### Acceptability and Feasibility

The primary outcome for this study was acceptability and feasibility of procedures for a trial and for the intervention. We recorded recruitment rates, the number of participants at each stage of the pilot study, reasons for attrition, randomisation errors and missing data for outcome measure at each time point. The time taken for assessment and the number and duration of follow up telephone calls per patient were recorded.

### Outcomes

We explored two potential primary outcomes for a definitive trial: depression and chest pain.

#### Depression

HADS-D[20] scores were used to calculate response (≥50% decrease in score from baseline at follow up), remission (score < 8 at follow up) and severity (continuous score). We also explored the PHQ-9[21] as an alternative measure of depression severity and extracted the number of GP/PN consultations for depression, antidepressant prescriptions and referrals to talking therapy during the 12 month study period from participants' medical records.

#### Chest pain

Self reported chest pain was measured using the Modified Rose Angina Questionnaire[19]. The number of GP/PN consultations for heart-related problems during the 12 month study period was extracted from participants' medical records as proxy measure of participants' cardiac status.

Potential secondary outcomes explored were: anxiety, (HADS-anxiety subscale)[20], well being (Warwick-Edinburgh Mental Well-being Scale)[22], quality of life (Short Form-12 (SF-12))[23], functional status (Specific Activity Schedule)[24], number of reported social problems (social problems questionnaire)[25], adherence to antidepressant medication (if relevant - Adapted version of Morisky Adherence Index)[26], and patient reported problems and needs (PSYCHLOPs)[27].

Self efficacy was measured using the General Self Efficacy Scale (GSES)[28] and illness beliefs using the Brief Illness Perceptions Questionnaire (BIPQ)[29]. The latter assesses changes in perceptions about illness along the following dimensions: Consequences, Timeline (anticipated duration of illness), Personal control, Treatment Control, Identity (symptoms associated with the illness), Illness Concern, Illness Coherence (understanding of CHD) and Emotional Representations (emotional impact of CHD).

The types of needs and problems identified by PC group patients in collaboration with their nurse-case manager as contributing to depression was extracted from nurse-case manager notes made during consultations. The BIPQ asks participants to “Please list in rank-order the three most important factors that you believe caused your illness” we also explored these responses.

Baseline demographic data including gender, age, ethnicity, socio-economic status (Index of multiple deprivation)[30], employment and relationship status, living arrangement and cardiac risk factors (smoking status, alcohol consumption, body mass index) were recorded.

### Costs of PC

Quality-adjusted life years (QALYs) were measured using the EQ-5D[31]; UK values were applied to the derived health states to estimate the utility value for each patient at each time point[32]. Economic costs were calculated from a healthcare plus informal care perspective. PC costs included the time spent by practice nurses with patients in face-to-face assessments and subsequent telephone reviews. A unit cost of £36 per hour was attached to the average intervention duration for each patient. Other service use was recorded using the Client Service Receipt Inventory[33] for the 6-month period preceding baseline, and 6- and 12-month follow-ups. Health services included hospital inpatient and outpatient visits, GPs, psychiatrists, psychologists, physiotherapists, counsellors, nurses and other therapists. Unit costs were applied to service use data using the NHS reference costs in 2009-10 prices[34] and the 2010 Unit Costs of Health & Social Care[35]. In addition, data were collected on the weekly number of hours of help (i.e. personal or child care, help in/around and outside the house) received from friends and relatives of the patient. The unit cost of a home care worker was used as a proxy for costing informal care.

### Sample size

Estimation of an effect size was not the focus of this pilot study. We aimed to recruit 80 participants (40 per arm) into the pilot study, we considered, based on past experience of recruitment from this population, that this would be feasible in the time available. We estimated from the results of the UPBEAT cohort study[9] that 10-15 practices each with around 10,000 patients would be needed.

### Randomisation

Randomisation at patient level was conducted independently by the Mental Health and Neurosciences Clinical Trials Unit (CTU) at King's College London. A random permuted block design was used to balance the numbers between groups. PC group participants were randomly allocated to one of two nurse researchers acting as case-managers.

### Blinding

Participants were asked at the beginning of each follow up interview not to mention whether they had been in contact with other study staff. The statistician was also kept uninformed of allocation status.

### Statistical analyses

Analyses were exploratory and were conducted using STATA verson 11[36]; the intention to treat principle was used. Descriptive statistics were used to describe the characteristics of the sample and the acceptability and feasibility data.

We developed a single statistical model to estimate the difference in mean scores between participants randomised to PC and TAU across the three follow-up points (one, six and twelve months). A linear mixed effect model for longitudinal data (random intercept model) was used to estimate (using maximum likelihood) the difference between treatment arms in scores at 1, 6 and 12 months overall. The assumption of normality for the residuals was checked visually from probability plots. The pre-specified covariates that were included in the model consisted of the baseline outcome score and the randomisation group. Time was included to estimate the time trends over the whole sample. An interaction between time and intervention was also examined for evidence of a differential effect over time. All tests of hypotheses were two-tailed and associated 95% Confidence Intervals (CI) are reported; due to the exploratory nature of the analysis, p-values are reported for the preliminary primary outcome (HADS-D) only.

The median number of responders (≥50% decrease in score from baseline at follow up) and remitters (score <8 at follow up) according to the HADS-D score was compared between groups using chi-square tests. We compared mean scores for self efficacy and illness perceptions between groups using independent two-sample t-tests. We controlled for the effects of self efficacy and illness perceptions on changes in depression severity using a linear mixed effect model for longitudinal data (random intercept model) to estimate (using maximum likelihood) the difference between treatment arms. We repeated these analyses to explore nurse (therapist) effects.

We used multiple regression analysis to estimate mean differences in costs and QALYs. Baseline and follow-up data were used as the dependent variables and the group identifier as an independent variable. The models for follow-up costs and QALYs were adjusted for baseline costs and utility scores. Cost-effectiveness comparisons were made using incremental cost-effectiveness ratios (ICERs) and the net benefit (NB) approach. incremental cost-effectiveness ratios were calculated by dividing the difference in mean total costs between the PC and TAU groups by the difference in mean QALYs. Net Benefit from the intervention to society was defined as the product of the willingness-to-pay for a QALY (within a range of £0 to £60,000) and the actual QALY gain minus the service costs (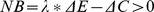). Net benefits were then compared between the PC and TAU groups for each willingness-to-pay value. Missing utilities at follow-up were imputed using the last value carried forward (LVCF) method[37].

To account for the highly skewed distribution of the cost data, the non-parametric bootstrap method was used to make cost comparisons between the two groups[38]. Bootstrapping involved repeatedly estimating the incremental cost-effectiveness ratio to account for the uncertainty surrounding the estimates of costs and benefits. Likewise, estimates of the proportion of iterations in which the intervention of interest had the maximum expected net benefit (NB), or equivalently, a positive incremental net benefit (INB) was determined for a range of willingness-to-pay thresholds. The estimates were produced by repeatedly sampling with replacement (1000 times) from the existing pilot study population[39]. The results of the bootstrap analyses were plotted on a cost-effectiveness plane (CEP) and used to estimate cost-effectiveness acceptability curves (CEACs). CEACs show the probability that each of the treatment options is optimal, subject to a range of ceiling ratios, which represent the maximum amount society would pay for a one unit improvement in QALYs[39].

## Results

17 practices were approached by the Greater London Primary Care Research Network (PCRN) and agreed to participate. Practices were recruited between October 2010 and June 2011; practice recruitment was therefore completed in considerably less than the 12 months planned in the study proposal indicating that recruitment of practices for a definitive trial would be feasible. Data collection was completed by July 2012.

Participant recruitment is detailed elsewhere[16]. In summary, 3325 persons on the 17 GP CHD registers, 1001 consented to be contacted. 126 were eligible for assessment (PHQ2 score ≥3 and reporting current chest pain). Of the 126, 40 had a HADS score 8, 2 had experienced hallucinations, 2 had no current chest pain and 1 did not have sufficient English. Following interview, 81 were found to be eligible, consented and randomised (41 to PC, 40 to TAU). Recruitment of patients for a definitive RCT therefore seems promising.

Fifty-two male and 29 female patients were enrolled. Ages ranged 38 to 95 years (mean 65 *SD*11), 83% were white. Forty-eight participants reported having ever been diagnosed with depression (21/41 = 51% PC, 27/40 = 67% TAU) of these 12 had had one episode, 7 had had two episodes, 4 had had three episodes, and 23 reported having had 4 or more episodes (data on number of episodes were missing for 2 participants). Forty-six participants had previously received treatment for depression; of these 41 had taken antidepressants and 29 had had talking therapy. Eighteen reported having received other treatment such as ‘anger management’, seeing a psychiatrist, ECT, inpatient psychiatric care and relaxation and assertiveness courses. Our participants therefore represent a chronic and severe group.

Twenty-four participants reported that they were currently receiving treatment for depression (9 in PC, 22%, 15 in TAU, 38%). According to the medical notes data, 13 in PC (32%) and 17 in TAU (43%) were taking some form of antidepressant medication at baseline. Despite being prescribed antidepressants, these participants were still reporting depressive symptoms. Nineteen participants reported their current episode had lasted more than 12 months, 2 said it had lasted between 6 and 12 months, and 3 said it had lasted less than 6 months.

Mean baseline HADS-D scores (PC 12, *SD*3; TAU 11, *SD*3) indicated moderate depression and mean PHQ9 scores (PC 16, *SD*5; TAU 15, *SD*6) indicated moderately severe depression in both groups. At baseline, according to the HADS-D, 21 (51%) participants in the PC group could be considered mild, 14 (34%) moderate, 6 (15%) severe; in the TAU group there were 19 (47%) mild, 15 (37%) moderate, 6 (15.0%) severe. For the PHQ9, 3 (7%) were mild, 10 (24%) moderate, 14 (34%) moderately severe, 12 (29%) severe in the PC group and 8 (20%) were mild, 8 (20%) moderate, 14 (35%) moderately severe, 9 (22%) severe in the TAU group. The correlation between baseline HADS D and PHQ9 was r  = 0.48.

### Baseline characteristics of intervention (PC) and control (TAU) groups

Participants in each group had similar mean age, mean years in education and mean BMI score compared. The PC group had more males, people of non-white ethnicity and people in paid employment compared to the TAU group. The TAU group had more females, more non-drinkers and more people in this group lived alone compared to the PC group. Those randomised to PC had marginally greater IMD scores than those in the TAU group indicating slightly higher levels of deprivation. However differences between the groups appeared small. Due to an oversight, 3 patients who were ineligible due to no current chest pain were randomised in error (2 in the PC group). Based on the intention to treat principle these were included in all analyses; a sensitivity analysis found that our conclusions were unaffected when these patients were omitted from the analyses. Table 1 shows the baseline demographic and lifestyle data by group and Table 2 shows baseline scores for all outcome measures by group. The groups appear to be balanced indicating that our randomisation process was successful.

**Table 1:** Baseline characteristics by group.

		PC (n = 41)		TAU (n = 40)	
Sociodemographic characteristics		n		n	
Gender	Male	27	66%	25	63%
Age (years) (Mean (*SD*))		64.2	13.0	64.9	8.5
Ethnicity	White	33	81%	34	85%
	Black	1	2%	2	5%
	Asian	2	5%	3	8%
	Other	5	12%	1	3%
IMD (Mean (*SD*))^1^		27.1	13.7	25.0	13.7
Years in education (Mean (*SD*))		11.7	4.1	12.3	3.7
Employment status^2^	Paid employment	8	20%	4	10%
	Retired	26	63%	29	73%
	Housewife/husband	2	5%	2	5%
	Unemployed/student	3	7%	4	10%
Relationship status	Married	21	51%	19	48%
	Cohabiting	5	12%	3	8%
	Widowed	5	12%	7	18%
	Separated	3	7%	1	3%
	Divorced	5	12%	6	15%
	Single/non cohabiting partner	2	5%	4	10%
Live with	Spouse	16	39%	12	30%
	Spouse and child(ren)	8	20%	8	20%
	Child(ren)	4	10%	3	8%
	Alone	12	29%	16	40%
	Other	1	2%	1	3%
Place of residence	Owner occupied house/flat	19	46%	14	35%
	Privately rented house/flat	2	5%	6	15%
	House/flat rented from local authority	18	44%	17	43%
	Sheltered housing/warden control	1	2%	3	8%
	Other	1	2%	0	0%
Cardiovascular risk factors					
BMI category^3^	Underweight	2	5%	1	3%
	Normal	9	22%	10	25%
	Overweight	10	24%	13	33%
	Obese	17	42%	13	33%
Smoking status	Never	12	29%	10	25%
	Ex	19	46%	21	53%
	Current	10	24%	9	23%
Average alcohol units consumed	Doesn't drink	17	41%	22	55%
	1–10	20	49%	9	23%
	Greater than 11	4	10%	9	23%
Self reported high cholesterol^4^	yes	21	62%	21	55%
Self reported hypertension^5^	yes	29	78%	27	69%
Self reported Diabetes^6^	yes	12^4^	30%	10	25%

1 IMD – index of multiple deprivation; Data are missing for:

2 2 participants in PC and 1 in the TAU;

3 3 participants in each arm;

4 7 participants in PC and 2 in TAU;

5 4participants in PC and 1 participant in TAU;

6 1participant in PC.

Percentages are rounded to the highest whole number and so may appear to indicate >100%

**Table 2:** Baseline scores for the preliminary primary and secondary outcome measures by group.

Measure		PC group n = 41	TAU group n = 40
		Mean (SD)	Mean (SD)
Severity of depression (HADS-D)***		11.6 (3.3)	11.4 (3.0)
Severity of depression (PHQ9)		16.0 (5.3)	15.4 (5.5)
Self-Efficacy (Self Efficacy Scale)*		26.1 (7)	27.7 (6.3)
Consequences (BIPQ1)		5.4 (3.1)	6.1 (3.2)
Timeline (BIPQ2)		9.3 (2.1)	9.2 (2.5)
Personal control*(BIPQ3)		3.5 (3.3)	3.5 (3.3)
Treatment control*(BIPQ4)		7.0 (2.9)	6.9 (2.9)
Identity (BIPQ5)		5.1 (8.1)	5.5 (3.2)
Illness concern (BIPQ6)		6.4 (3.7)	6.1 (3.9)
Illness coherence* (BIPQ7)		6.0 (4.1)	5.7 (3.6)
Emotional representations (BIPQ8)		6.7 (3.4)	6.1 (3.5)
Severity of Anxiety (HADS-A)		12.6 (4.6)	12.6 (5.2)
Well being (WEMWBS)*		37.6 (9.7)	34.8 (9.6)
QoL physical (SF12 Physical component)*		31.9 (9.9)	33.8 (10.1)
QoL mental (SF12 Mental component)*		28.7 (9.2)	28.3 (8.4)
Patient-generated measure (Psychlops)		15.6 (4.0)	16.1 (3.4)
		N (%)	N (%)
Chest pain (modified Rose Angina questionnaire)	No	2 (5)**	1**(2)
	Yes	39 (95)	39 (98)
Functional status (Specific Activity Schedule)	1	13 (32)	12 (30)
	2	6 (14)	6 (15)
	3	16 (40)	13 (33)
	4	6 (14)	9 (23)
Adherence to medication (Morisky Index)	High	11 (33)	20 (57)
	Intermediate	20 (61)	14 (40)
	Low	2 (6)	1 (3)
Number of social problems (SPQ)	0	9 (22)	6 (15)
	1	4 (10)	11 (28)
	2	9 (22)	7 (18)
	3	7 (17)	9 (23)
	4–8	12 (29)	7 (18)

*high score  =  better.

**randomized in error, having chest pain was an inclusion criterion.

***HADS-D ≥8 was an inclusion criterion.

### Procedures and participation

**Figure 1:**
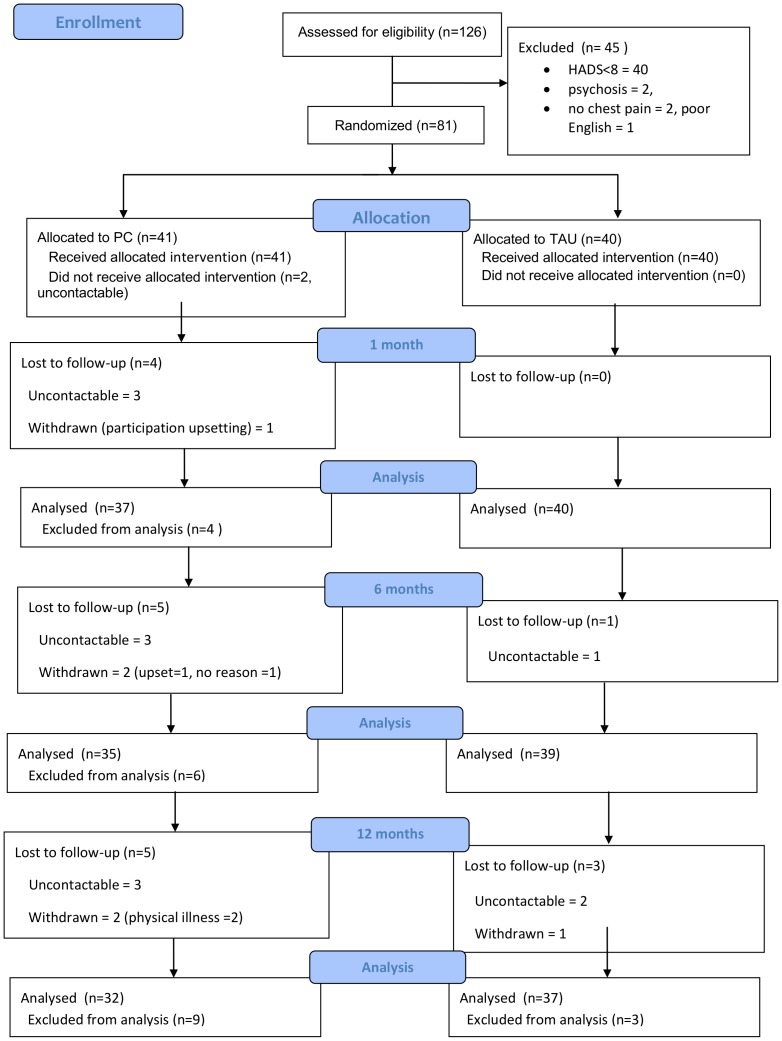
UPBEAT pilot study Consort diagram. Uncontactable means lost to follow-up.

The consort diagram for the pilot study is shown in Figure 1. By 12 months, 6 people in the PC group had dropped out (2 because they found participation upsetting, 2 because they felt too physically unwell to continue, 2 gave no reason) and 1 from the TAU group had dropped out (because they found participation upsetting). Two PC group participants received baseline assessment but no intervention as the nurses were subsequently unable to contact them. Overall, attrition was low (7/81 = 9%), with data collected at one or more follow up points for 79 people (98%).

### Data collection

The maximum number of observations available was 81 at baseline, 77 at 1 month, 74 at 6 months and 69 at 12 months. Two of our outcome measures had no missing scores at any point: the modified Rose Angina Questionnaire and the Specific Activity Schedule. The BIPQ had the most missing scores, with 14% missing at 6 months. At one or more assessment points, between 5% and 10% of scores were missing for the General Self Efficacy Scale, the BIPQ (items 3 and 4), the WEMWBS; all other measures had fewer than 5% of scores missing at any time. Therefore, these outcome measures appear to be acceptable to participants and would be feasible to use in definitive trial.

### Changes in outcomes

#### Depression severity

Both groups showed improvement in depression symptoms (Tables 2 and 3)) at all time points. Mean HADS-D score in both groups moved from indicating moderate depression at baseline to mild depression at 12 months; mean PHQ9 scores in both groups indicated moderately severe depression at baseline reducing to moderate depression at 12 months.

**Table 3:** Mean scores (SD) for outcomes at 1, 6 and 12 months and (where applicable) mixed effects model.

		1 month follow up		6 months follow up		12 months follow up		Mixed effects model (where calculated)
		PC	TAU	PC	TAU	PC	TAU	
Depression (HADS-D)		11.0 (3.4)	10.0 (4.5)	10.3 93.8)	9.2 (4.6)	9.5 (4.6)	8.8 (4.8)	−0.73 (−2.08, 0.62)†
Depression (PHQ9)		14.8 (6.4)	13.0 (6.8)	13.4 (7.0)	11.7 (6.5)	12.6 (7.1)	12.0 (6.9)	−0.63 (−2.60. 1.35)†
Chest pain (yes) (Modified Rose Angina Questionnaire)		27 (73%)	29 (75%)	22 (63%)	32 (82)†	22 (69%)	30 (81%)	2.21 (0.69, 7.03)††
Self Efficacy (GSES)*		27.7 (7.0)	27.1 (6.3)	26.7 (8.1)	28.2 (6.9)	28.6 (6.7)	27.9 (8.1)	−0.58 (−3.05, 1.89)†
Illness perceptions (BIPQ total score)		42.8 (13.3)	44.4 (13.3)	43.0 (13.3)	40.9 (11.5)	40.0 (14.8)	43.0 (13.1)	−0.42 (−4.57, 3.72)†
Anxiety (HADS –A)		12.2 (4.3)	11.3 (5.0)	11.0 (5.0)	10.4 (5.0)	9.9 (4.9)	9.5 (5.4)	−0.80 (−2.08, 0.48)†
QoL (SF12 Physical)*		31.6 (8.4)	32.8 (9.3)	31.3 (10.6)	31.7 (9.1)	32.4 (10.7)	33.3 (9.2)	−0.04 (−2.85, 2.77)†
QoL (SF12 Mental)*		31.4 (10.8)	30.8 (11.0)	32.4 (11.2)	35.4 (11.8)	34.5 (11.6)	33.6 (12.5)	1.27 (−2.01, 4.55)†
PSYCHLOPS		14.6 (4.4)	14.8 (4.9)	13.1 (5.4)	14.0 (5.9)	13.6 (5.1)	13.4 (5.4)	−0.39 (−2.20, 1.42)†
Well being (WEMWBS)*		37.5 (11.1)	36.1 (10.4)	38.4 (12.2)	39.6 (11.6)	40.6 (11.2)	39.6 (12.3)	1.59 (−1.98, 5.15)†
Morisky adherence Index	High	8 (29%)	17 (52%)	12 (57%)	13 (46%)	8 (38.1%)	15 (60%)	
	Intermediate	19 (68%)	13 (39%)	9 (43%)	12 (43%)	12 (57%)	10 (40%)	
	Low	1 (4%)	3 (9%)	0 (0%)	3 (11%)	1 (5%)	0 (0%)	
Functioning (Specific Activity Schedule)	1	10 (27%)	16 (40%)	9 (26%)	10 (26%)	6 (19%)	8 (22%)	
	2	8 (22%)	2 (5%)	7 (20%)	7 (18%)	7 (22%)	7 (19%)	
	3	15 (41%)	16 (40%)	15 (43%)	18 (46%)	12 (38%)	17 (46%)	
	4	4 (11%)	6 (15%)	4 (11%)	4 (10%)	7 (22%)	5 (14%)	
Social Problems Questionnaire (SPQ total N problems)	None	11 (26.8)	3 (7.5)	15 (36.6)	8 (20.0)	16 (39.0)	9 (22.5)	
	One	7 (17.1)	9 (22.5)	10 (24.4)	13 (32.5)	8 (19.5)	13 (32.5)	
	Two	13 (31.7)	8 (20.0)	5 (4535)	4 (10.0)	5 (12.2)	8 (20.0)	
	Three	3 (7.3)	8 (20.0)	5 (12.2)	8 (20.0)	5 (12.2)	5 (12.5)	
	Four-Eight	7 (17.1)	12 (30.0)	6 (14.6)	7 (17.5)	7 (17.1)	5 (12.5)	

*a high score is good. See Table 2 for baseline values,

† mean difference (95% CI),

†† odds ratio (95% CI).

#### Depression remission (HADS-D)

There was a greater percentage of remitters in the TAU (36%, 14/39) compared with the PC group (24%, 8/34) at 6 months, and at 12 months (TAU: 41%, 15/37 *versus* PC 34%, 11/32).

#### Depression response

At 6 months there was greater percentage of responders in the TAU (21%, 8/37) compared with the PC group (15%, 5/34), but by 12 months more PC group participants had responded (TAU  = 24%, 9/37, PC  = 28%, 9/32).

However, the mixed effects models showed no significant differences between groups over time for any measure of depression and confidence intervals were wide so an effect in favour of either group cannot be ruled out (PC-TAU severity: mean diff −0.73, 95% CI −2.08, 0.62, p = 0.29); remission: OR 2.67, 95% CI 0.71, 10.4, p = 0.15; response: OR 1.33, 95% CI 0.38, 4.61, p = 0.65).

#### Receipt of depression treatment (medical notes)

Across the 12 month pilot study period, in the PC group, 31 participants (76%) saw their GP or PN regarding their mental health (total of 101 consultations recorded); in the TAU group, 29 participants (73%) made a mental health consultation (total of 102 mental health consultations recorded). Of those participants who were not treated for depression at baseline (i.e. no record of antidepressant prescription or talking therapy referral), 3 PC participants had received a prescription for an antidepressant (Citalopram ×2, Mirtazepine ×1, one of these participants was also referred for ‘counselling’) and 1 additional PC group participant had been referred to a ‘psychiatric clinic’ by 12 months; no participants in the TAU group had a new referral for depression treatment or a new prescription for an antidepressant at the end of the pilot study.

#### Chest pain

**Figure 2:**
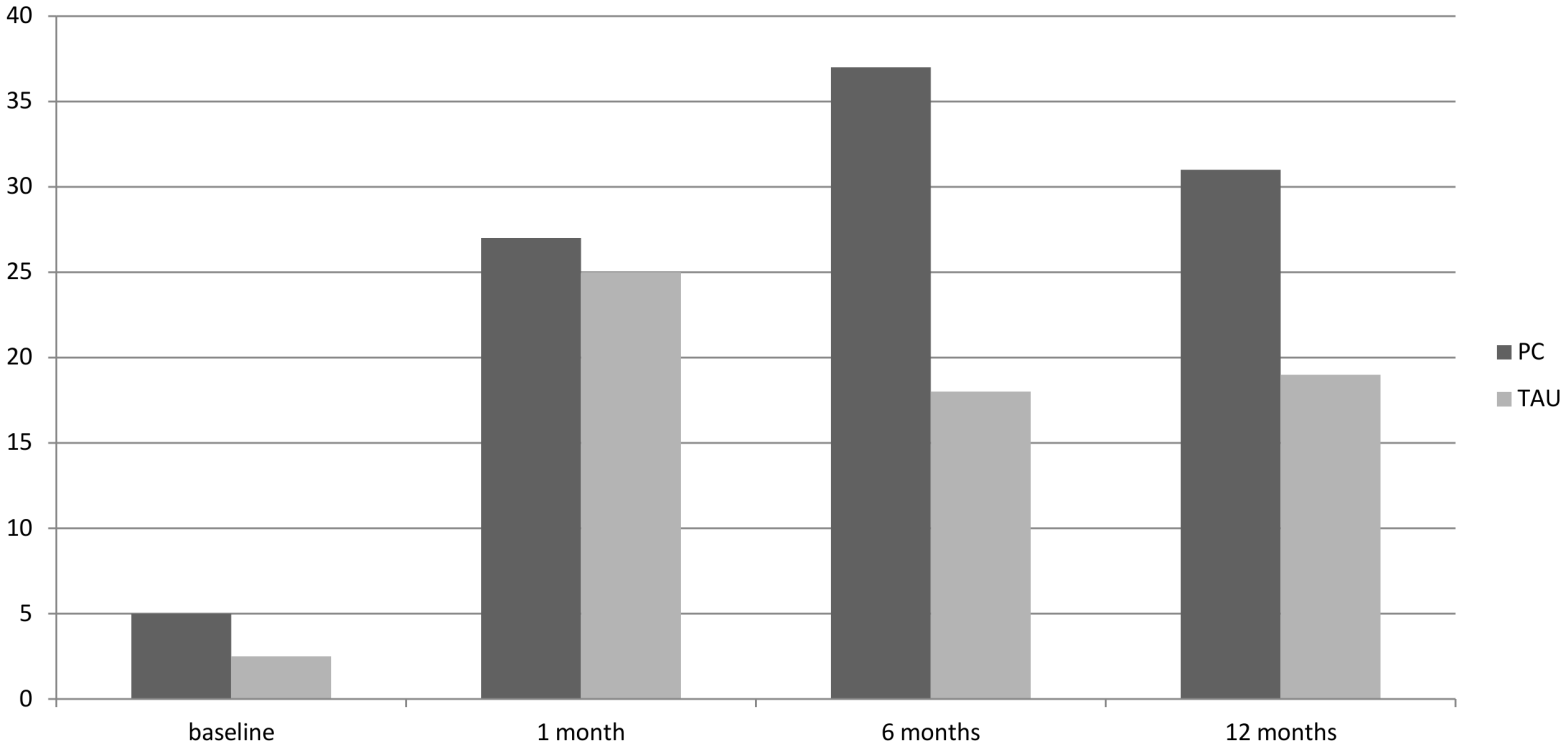
Changes in self reported chest pain as reported using the modified Rose Angina Questionnaire. % =  those reporting no chest pain at each time point. OR TAU vs PC: 2.21 95% CI 0.69, 7.03.

At 6 months the proportion of patients who no longer reported chest pain (Modified Rose Angina Questionnaire, Figure 2) was 37% in the PC group *versus* 18% in the TAU group and at 12 months it was 31% in the PC group *versus* 19% in the TAU group. From the medical notes across the 12 month pilot study period, in the PC group, 34 participants (83%) saw their GP or PN regarding their CHD (total of 158 consultations recorded); in the TAU group, 29 participants (73%) made a CHD consultation (total of 170 consultations recorded); it was unclear whether these were routine or emergency visits.

A&E attendances over the 12 month study period were identified from medical notes. Ten PC group participants (6 for heart problems, 2 for other state reasons, 2 no reason recorded) visited A&E (total 13 visits: 9 heart problems, 2 other stated reasons, 2 no reason recorded); 15 TAU group participants (4 for heart problems, 5 for other state reasons, 6 no reason recorded) visited A & E (total 26 visits: 7 heart problems, 6 other stated reasons, 13 no reason recorded). PC participants therefore made fewer A&E visits (24% in PC versus 38% in TAU), although missing data concerning the reason for these visits makes this information difficult to interpret.

#### Preliminary secondary outcomes

At 6 and 12 months both groups improved on all outcomes (Table 3). There was no evidence for an interaction between time point and study arm for any outcome.

### Appropriateness of study outcomes

All participants were asked to list the 3 most important problems which they felt had caused their CHD; 61 gave at least one reason. The most common reason was ‘genetics or heredity’, followed by lifestyle factors such as smoking, poor diet and lack of exercise. Mood problems, especially stress and work-related stress were also mentioned, and co-morbid or past health problems were also blamed. Four patients mentioned relationship problems and 1 mentioned financial problems (a full list of responses is given in Appendix S1 online).

Participants in the PC group (N = 41) identified 21 types of problem as contributing to their depression and which were addressed during the intervention (up to 3 problems per patient); most common were (patients): pain (chest and other pain e.g. arthritis) (18), lack of exercise (17), difficulty sleeping (13), anxiety (11), overweight (11). Reported problems and whether or not they were addressed during the intervention are listed in Appendix S2 online.

Participants therefore explained both their CHD and their depression in terms of wide ranging problems; lifestyle and mood problems, in particular, were commonly associated with both. Within our pilot study, mood outcomes were assessed using the HADS and the PHQ9, however we had no measure of change in lifestyle-related outcomes. The PC intervention was aimed at tackling the problems that each participant felt were important rather than addressing specific cardiac risk factors as has been tested in other trials, however it appears that patients consider CHD-related lifestyle factors to contribute to their depression, so inclusion of a measure of these factors should be considered for a definitive trial of PC. It will be important to select a measure which captures the variation between participants in terms of which risk factors they want to address; a validated measure of goal attainment may therefore be appropriate.

### Intervention procedures

There was considerable variation between patients in the number of failed follow up contact attempts by nurses over the 6 month intervention period (range: 0 to 32), but on average nurses made 2.8 calls for every successful contact. PC participants (n = 41) received a mean 203 SD100 mins of nurse time (78 SD19 for assessment; 125 SD91 in telephone follow up calls) over 6 months. Mean number of follow up calls was 9 SD5; the mean duration of calls was 14 SD4 minutes.

### Mediator Analyses

#### Self-efficacy for self management and illness perceptions

At 12 months, the PC group had a mean increase in self-efficacy of 2.5 points *versus* 0.9 points in the TAU group. Similarly, mean improvement in overall illness perceptions score was greater in the PC compared with the TAU group: 7.8 points *versus* 2.5 points. The biggest difference in mean improvement between the PC and TAU groups was in “Personal Control” (mean change in BIPQ from baseline  = 1.1 for PC *versus* 0.1 for TAU at 12 months). Controlling for changes in self efficacy or overall illness perceptions had little effect on change in depression over time, whether considering depression (mean diff, 95% CI) severity (self-efficacy −0.88, 95% CI −2.11, 0.36; illness perceptions −0.57, 95% CI-1.9, 0.77), remission (OR) (self-efficacy 3.15, 95% CI 0.64, 15.50; illness perceptions 3.26, 95% CI 0.94, 11.24) or response (OR) (self-efficacy 1.02, 95% CI 0.26, 3.95; illness perceptions 1.27, 95% CI 0.34, 4.66).

#### Anxiety

Since anxiety symptoms were high at baseline, in a *post hoc* analysis, we explored HADS-A score as mediator for improvement in depression. Controlling for anxiety slightly reduced the difference in depression symptoms between the groups over time: mean difference  = −0.43 95% CI −1.48, 0.63, p = 0.43. Controlling for anxiety considerably reduced the odds of remission in the TAU versus PC group in favour of the PC group: OR remission in TAU *versus* PC group  = 0.42, 95% CI 0.10, 1.68, p = 0.22 which suggests that changes in anxiety symptoms may be a mediator for depression remission. The odds of depression response in the TAU group compared with the PC group were also slightly reduced when anxiety scores were controlled, although the odds were still in favour of TAU: OR 1.12 95% CI 0.32, 3.9.

#### Therapist effects

The random effects model (combining data from 1, 6 and 12 months) indicated little difference in the average therapist effect on the HADS-D score across the time points (adjusting for baseline HADS depression score): mean difference  = −0.86 (−2.81 to 1.10).

Regarding self reported chest pain, of Nurse 1's (registered general nurse and health psychologist) patients, 44% continued to report chest pain at 6 months compared with 79% of Nurse 2's (registered general and mental health nurse) patients at 6 months (p = 0.03). In the random effects model, the odds of reporting chest pain across the study period were higher for Nurse 2 compared with Nurse 1: OR  = 7.80 (0.88 to 69.40). Therefore, a therapist effect cannot be ruled out.

#### Intervention intensity

The amount of time spent talking to the nurse varied considerably between patients (range 74 to 406 minutes), so we used the median duration (167 minutes) to divide the participants into high (n = 20) and low (n = 19) ‘dose’ groups. There were no significant differences (p>0.05) between the groups at baseline in depression (HADS-D mean: low dose group 11.0 *SD* 3; high dose group 12 *SD* 3.7). The magnitude of improvement in depression over time was greater for the high compared to the low dose group and fewer high dose patients had chest pain at 6 and 12 months, although the mixed effects models indicated little difference between the groups: depression mean difference −0.72 95% CI −3.03 to 1.60; chest pain OR 0.34, 95% CI 0.01 to 7.53.

### QALY gains

The average EQ-5D utility scores at baseline were slightly higher for the PC group (see Appendix S3), although the difference between groups was not statistically significant (95% CI −0.98 to 0.25, *p* = 0.40). By the 1-month follow-up the TAU group had a higher utility score and this difference was maintained up to 12-month follow-up (95% CI 0.26 to 0.11, *p* = 0.422, at 6-month follow-up: 95% CI 0.27 to 0.11, *p* = 0.408). In terms of QALYs, the control group showed an incremental QALY gain of 0.038 compared to personalised care over the 12-month treatment period.

#### Service use and costs

Service use was fairly similar between the intervention and the control groups during the study period (Total cost PC *vs* TAU, mean (SD): baseline 1,773 (2,498) *vs* 3,604 (7,852); 6 months 832 (1,383) *vs* 1,191(1,168); 12 months 1,088 (1,320) *vs* 2,014 (3,246); details are shown in Appendix S4). Hospital services were used more intensively by the TAU group than the PC group at all time points, with inpatient and outpatient care being the most frequently used services. The TAU group incurred higher inpatient costs than the PC group at each time point (particularly baseline and at 12-month follow-up). Few patients used day hospital services, but the costs incurred were high for both groups. The majority of patients received care from GPs and the costs of this were similar between the groups.

Informal care was used slightly more among patients in the PC group compared with the TAU group. Average total costs at each time point were lower for the PC group compared to the TAU group. However, the differences were not statistically significant. For the PC group, the intervention itself accounted only for 6.7% of total costs.

#### Cost-utility analysis

Of the total 81 participants, cost and QALY data at each time point were available for 68 patients (84%). Cost-utility results yielded an incremental cost-effectiveness ratio (ICER) of £29,921 per additional QALY. Cost-effectiveness plane and cost-effectiveness acceptability curves (CEACs) were produced from bootstrapped resamples. The distribution of the cost-effectiveness point estimates on the cost-effectiveness plane (Appendix S5) indicated a strong likelihood of cost savings for the PC group compared with the TAU group. The point estimate of the incremental cost-effectiveness ratio falls in the south-western (SW) quadrant, representing the situation where the PC group has reduced costs and worse outcomes. The second most likely result is that PC results in lower costs and better outcomes (SE quadrant).

**Figure 3:**
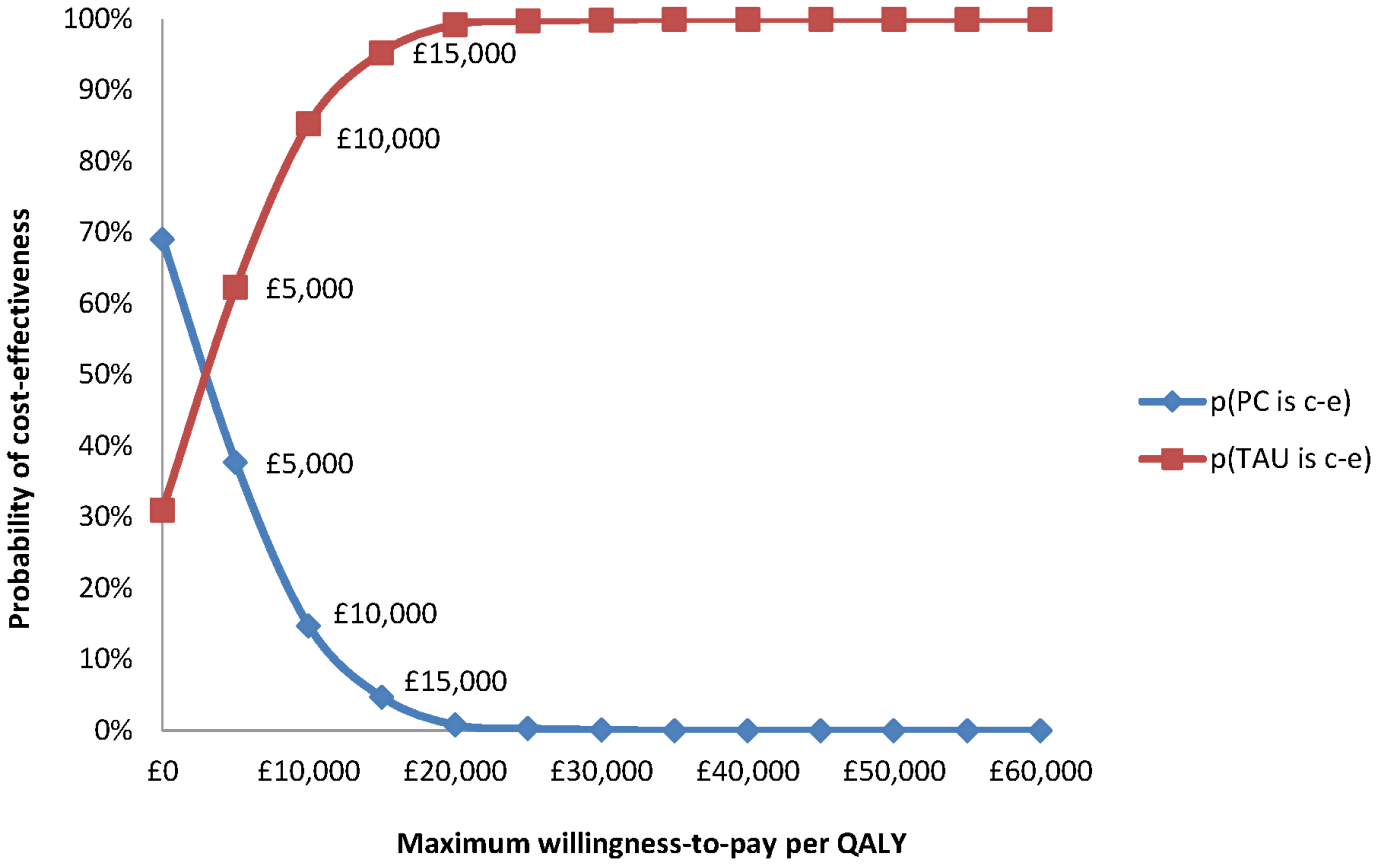
Cost-effectiveness acceptability curves (CEACs) showing the probability that each of the treatment options is optimal, subject to a range of ceiling ratios, which represent the maximum amount society would pay for a one unit improvement in QALYs.

The CEAC (Figure 3) for the PC group compared to the TAU group was downward sloping. There is a greater likelihood of PC being the most cost-effective option up to a QALY threshold of £3,035.

## Discussion

This study explored the feasibility and acceptability of trial and intervention procedures for an RCT of a practice nurse-delivered personalized care intervention for primary care CHD patients who have probable depression and current chest pain. We also examined the potential costs of the intervention.

### Feasibility and Acceptability of Personalised Care and Appropriateness of Study Outcomes

The PC intervention appeared to be feasible and acceptable for use in current primary care: little nurse time was needed, engagement with the nurse-case managers was high and drop out was low. The findings of our qualitative work[8], [13] that patients with CHD and probable depression report a wide range of problems which they consider contribute to their low mood were confirmed. The PC intervention enabled patients to identify and address these problems with the nurse-case managers. This is an improvement on current care for these patients since management of depression and/or psychosocial problems is not routinely addressed in this population[8]. Compared to the widespread organizational change which would be needed for collaborative care interventions as trialled in the USA[6], our personalized care intervention appears to offer an enhanced form of treatment as usual which could be implemented easily in current primary care practice. Evidence from the UK-based ProCEED trial[40] that care reviews delivered by practice nurses acting as case managers were acceptable to patients with long-term depression supports this[14].

We explored a wide range of outcomes focusing on depression as measured by the HADS-D as a potential primary outcome. Both groups improved on all measured outcomes, since depression and functional status outcomes changed in the same direction, we see little reason to change to a different primary outcome for a definitive trial. Our mixed effects model indicated that PC would be unlikely to do much harm compared with TAU (mean difference −0.73), but could improve symptoms up to 2.1 points on the HADS-D (95% CI −2.08, 0.62).

There were no large differences between groups, except in self reported chest pain, which was also an inclusion criterion for the study. We are unable to determine whether self-reported chest pain in our study participants was of cardiac origin. It is estimated that in half of all patients presenting with chest pain, the pain is of non-cardiac origin(i.e. no physiological cause can be identified)[41]. A systematic review (15 studies, 803 participants) [42] of psychological interventions for chest pain in patients with normal coronary anatomy suggests a modest to moderate benefit[43]. Our pilot study indicates that non-pharmacological intervention may also be effective for chest pain in patients with CHD. Our cohort study[15] indicated that chest pain has a range of negative impacts; chest pain therefore remains an important outcome for a future trial but self-report should be supported by a more objective measure of cardiac status.

As predicted, our data indicated that self efficacy and illness perceptions, especially personal control which is closely related to self efficacy, were increased in those receiving PC. A difference between our intervention and that of others which have not found improved self efficacy[44] following self management intervention is that our participants chose the outcomes on which to work, that isthey identified the factors they felt contributed to their low mood, rather than being required to work directly on their depression. A better examination of the theory behind our PC intervention would be to explore the effect of changes in self efficacy on a measure of goal attainment, then test the effects of goal attainment on depression over the long term, although this would require a complex trial. Fewer PC compared with TAU participants visited A&E (24% versus 38%), which may indicate increased self efficacy in self management in the PC group, though in a future trial a more robust measure than self report of A&E attendance should be used to examine this, for instance Hospital Episode Statistics (ref health and social care information center)[45].

### Acceptability and feasibility of the study protocol

Our findings suggest that the study protocol was feasible and acceptable. Attrition was below 10% and rates of missing data for most outcomes were low, despite the large number of measures. The Greater London Primary Care Research Network was responsible for practice recruitment which was achieved well within our predicted time-frame. Patient recruitment was in line with other studies of depression interventions in primary care[7], [46], [47]. Only around a third of patients invited to participate by their GP provided consent to contact. Use of this ‘opt in’ system appears to result in substantial loss of potential participants; however this is the usual method of recruitment for studies conducted in UK primary care. All of the patients meeting our inclusion criteria at baseline agreed to be randomised.

### Implications of clinical findings for a future trial of PC

An implication of the limited difference between PC and TAU and the small degree of change in depression symptoms detected over time, is that a sample size would be required (e.g. using the HADS-D mean PC 10.3 SD 4.6, TAU 9.2SD 4.6 at 6 months) of 368 per group for 90% power at a 5% significance level (two-sided). The sample size would be increased to some extent if a cluster design were employed, which would be necessary to reduce contamination if PC were tested using PNs based in practice.

To increase the expected effect size, the intervention could be given to a group that is more responsive. Our sample appears to represent a hard to treat group: the level of depression symptoms was high, more than half reported recurrent depression and more than a quarter reported that they were receiving depression treatment at baseline and yet still reported depression symptoms. In addition, we included only those patients with current chest pain and pain has been found to predict a worse course of mood disorders[48].

Alternatively, our finding that receipt of more nurse time was associated with greater improvement in depression and self reported chest pain suggests that more intensive intervention may be needed for the type of patient we recruited. Depression in older adults in primary care is increasingly conceptualized as a chronic illness[49]. In people with CHD[4], [50], even intensive treatments, such as CBT, problem solving and SSRIs have only a small effect on depression. Interprofessional working to ensure receipt of guideline informed treatment has been a key element in a number of successful trials of complex interventions for depression in primary care patients[6], [7], [51]. In this pilot study, four participants in the PC group compared with none in the TAU group received new depression treatment (antidepressant treatment or psychological treatment) by the end of the pilot study. The nurse-case managers contacted the GP or PN of the majority of PC group participants; however they reported difficulties: several telephone attempts needed, lack of response to emails, and even when contact was made, guideline informed treatment was not necessarily delivered (e.g. due to anxiety concerning multi-pharmacy and IAPT services being unavailable in some areas). Difficulties in implementing collaborative working for comorbid problems such as depression and CHD and uncertainties about the best service delivery approaches have been identified by other researchers[52]. In a future trial procedures for delivering guideline informed treatment should be pre-determined and case managers should be embedded within practices to increase interprofessional working (e.g. by having planned times for discussion of cases). In addition, in a future trial, more active treatment of anxiety should be tested. Anxiety is associated with worse depression outcome[53]; our participants reported high anxiety levels and we found some evidence of anxiety as a mediator for depression improvement.

The potential to detect differences between PC and TAU in our pilot study may have been reduced because TAU is itself an active intervention: at baseline 43% of the TAU group versus 32% of the PC group was prescribed antidepressants (according to their medical notes), we were unable to control for this difference in our analyses. It is also possible that TAU may have been intensified during the pilot study: during our qualitative work GPs and PNs reported greater awareness of the problem of co-morbid depression and CHD as a result of participation in our cohort study, the same may apply to participation in our pilot study, although from the medical notes it appeared that the number of mental health consultations were similar for both groups. In a future trial, changes in TAU during the trial should be recorded.

A PC manual (available from the authors) was produced and, in weekly study group meetings, the multidisciplinary clinical team was satisfied that the intervention was delivered as planned. Nevertheless our findings of potential therapist effects suggest that training in the behaviour change aspects of the intervention is important. However, other studies have shown that nurses and GPs trained in behaviour change techniques may have difficulty applying them[44], [54], [55]. Further research into how non-psychologists can be trained to work more psychologically is needed. Implementation of self management support interventions within primary care is known to be problematic[44]; a strength of our intervention compared with that of others[44]is the very short amount of nurse time required and the lack of need for specialist materials to be used or developed.

### Potential costs of PC

There were no great differences in service use and costs between PC and TAU, with the exception of inpatient care, which also accounted for a substantial proportion of total costs. Overall, it appears that PC reduced costs compared with TAU, but produced slightly lower benefits. However, costs may have been underestimated due to reliance on patient self-report in service use, the lack of medication and sick-leave data at all time points and the approach used to quantify informal care. Informal care constitutes a major cost driver in chronically ill populations. In this analysis, the “proxy good method” [56]and the unit cost of home care worker was used to calculate informal care. However, in a future trial, an alternative cost, such as the national minimum wage, could be used to quantify informal care in the context of a sensitivity analysis. A future trial should also test whether a longer time-horizon is needed for this particular patient group to benefit from an intervention of this kind.

## Conclusions

We have developed an intervention for primary care CHD patients with probable depression and current chest pain. We found that this intervention and our study protocol were feasible and acceptable for an RCT in this population. This pilot study was not powered to detect between group differences over time, but our data suggests that to be more effective case managers should be trained in behaviour change techniques, the intervention should be more focused on delivering guideline informed care, do more to address anxiety and include more intensive follow up. Collaborative working will be more feasible with case managers based within trial practices. The effects of differences in TAU will need to be considered in a future RCT. In addition, careful thought should be given to which primary outcome to use to reflect differences between patients in their desired outcomes, depression outcomes may only improve in the longer term. The UPBEAT personalized care intervention helps patients to address a wide range of problems which are not currently managed in primary care and appears to be cheaper than TAU.

## Supporting Information

Appendix S1 **The three most important factors that study participants felt caused their CHD.**All participants were asked to list the 3 most important problems which they felt had caused their CHD; 61 gave at least one reason. The question was asked as part of the Brief Illness Perceptions Questionaire (BIPQ).
(DOCX)

Appendix S2 **Problems reported by patients as contributing to their depression and whether or not the patient chose to address it during the intervention. Intervention group participants selected up to 3 problems to address during the intervention; some patients chose not to address a reported problem as part of the intervention.** Problems were categorised from the nurses' notes following the intervention; categories were agreed between the two nurses through discussion.
(DOCX)

Appendix S3 **EQ-5D score and QALY gain.**
(DOCX)

Appendix S4 **Service Use & Costs (£) at baseline, 6-month and 12-month follow-ups (by randomisation group).** Measured using the Client Service Receipt Inventory (CSRI).
(DOCX)

Appendix S5 **Distribution of the cost-effectiveness point estimates on the cost-effectiveness plane.**
(DOCX)

Checklist S1 **CONSORT Checklist.**
(DOC)

Protocol S1 **Trial protocol.**
(DOC)
