# Supplementary material for: The UPBEAT Nurse-Delivered Personalized Care Intervention for People with Coronary Heart Disease Who Report Current Chest Pain and Depression: A Randomised Controlled Pilot Study
Source: PLoS One. 2014 Jun 5;9(6):e98704. doi: 10.1371/journal.pone.0098704 (PMC4047012; doi:10.1371/journal.pone.0098704)
Supplement: Appendix S1 — The three most important factors that study participants felt caused their CHD.All participants were asked to list the 3 most important problems which they felt had caused their CHD; 61 gave at least one reason. The question was asked as part of the Brief Illness Perceptions Questionaire (BIPQ). (DOCX) [file pone.0098704.s001.docx]

**Appendix 1.** The three most important factors that study participants felt caused their CHD

| **Category** | **Patients’ own words as recorded by a researcher** | **Importance**  **(N participants)** | | |
| --- | --- | --- | --- | --- |
|  |  | **1st** | **2nd** | **3rd** |
| Genetic | Genetic/hereditary | 18 | 7 | 5 |
| Life style | Smoking | 13 | 8 | 1 |
|  | Poor diet | 4 | 6 | 3 |
|  | Over weight | 3 | 2 | 1 |
|  | Alcohol | 2 | 1 | 2 |
|  | Lack of exercise/sedentary lifestyle | 1 | 4 | 5 |
|  | Lifestyle/Poor diet plus lack of exercise/lifestyle when younger | 0 | 2 | 3 |
|  | Drug taking/cannabis/cocaine | 0 | 2 | 0 |
| Mood | Over work/work stress | 6 | 3 | 0 |
|  | Unhappy/depression | 1 | 1 | 0 |
|  | Stress/worry | 4 | 3 | 6 |
|  | Death of wife | 0 | 1 | 0 |
|  | Tiredness | 0 | 1 | 0 |
| Other medical problems | diabetes | 2 | 1 | 0 |
|  | cholesterol | 1 | 1 | 1 |
|  | High blood pressure | 1 | 0 | 0 |
|  | Pain in the chest | 1 | 0 | 0 |
|  | History of palpitations | 0 | 0 | 1 |
|  | History of pneumatic fever/weak heart | 0 | 0 | 1 |
|  | pneumonia | 1 | 0 | 0 |
|  | Operation for thrombosis | 1 | 0 | 0 |
|  | HRT implants | 0 | 1 | 0 |
|  | Side effects of HIV medication | 0 | 1 | 0 |
|  | Accident 1 year ago creating shoulder issue | 1 | 0 | 0 |
|  | Being run over early in life | 0 | 1 | 0 |
|  | Old age | 1 | 0 | 0 |
| Relationship problems | Matrimonial problems/divorce | 1 | 1 | 0 |
|  | Wife’s depression | 0 | 0 | 1 |
|  | Physical abuse | 0 | 0 | 1 |
| Financial problems | Financial problems | 0 | 1 | 0 |
| Idiosyncratic | Spider bite | 1 | 0 | 0 |
|  | Bad luck | 1 | 0 | 0 |
|  | looking after invalid husband caused strain | 1 | 0 | 0 |
|  | Radio pirates collapsed ceiling | 1 | 0 | 0 |
|  | Dust from mines | 1 | 0 | 0 |
|  | Working with asbestos | 0 | 1 | 0 |
|  | Living on the streets | 0 | 1 | 0 |
|  | Spraying cars without mask | 0 | 1 | 0 |
|  | Heavy lifting when in the Wrens | 0 | 0 | 1 |
|  | Overactive | 0 | 0 | 1 |
|  | Doesn’t know/no reason other than the one given/missing | 17 | 30 | 48 |

Question was asked as part of the BIPQ.
