# Supplementary material for: The UPBEAT Nurse-Delivered Personalized Care Intervention for People with Coronary Heart Disease Who Report Current Chest Pain and Depression: A Randomised Controlled Pilot Study
Source: PLoS One. 2014 Jun 5;9(6):e98704. doi: 10.1371/journal.pone.0098704 (PMC4047012; doi:10.1371/journal.pone.0098704)
Supplement: Appendix S2 — Problems reported by patients as contributing to their depression and whether or not the patient chose to address it during the intervention. Intervention group participants selected up to 3 problems to address during the intervention; some patients chose not to address a reported problem as part of the intervention. Problems were categorised from the nurses' notes following the intervention; categories were agreed between the two nurses through discussion. (DOCX) [file pone.0098704.s002.docx]

**Appendix 2.** Problems reported by patients as contributing to their depression and whether or not the patient chose to address it during the intervention

| **Problem defined by patient as contributing to depression** | **Addressed during intervention**  **(patients)** | **Reported by not addressed during intervention (patients)** |
| --- | --- | --- |
| Using cannabis | 2 |  |
| Anxiety | 11 |  |
| Sensory | 4 | 3 |
| Medication | 6 | 1 |
| Sexual | 2 | 3 |
| Breathing | 2 | 5 |
| Sleep | 13 | 11 |
| Pain (chest and other) | 18 | 7 |
| Alcohol | 2 |  |
| Smoking | 5 | 6 |
| Weight | 11 | 8 |
| Exercise | 17 | 8 |
| Daily activity | 8 | 7 |
| Mobility | 9 | 3 |
| Housing | 4 | 3 |
| Loneliness | 3 | 2 |
| Relationship | 5 | 4 |
| Financial | 3 | 1 |
| Caring responsibility | 2 | 4 |
| Employment | 7 | 1 |
| Education | 3 |  |

Patients selected up to 3 problems to address during the intervention; some patients chose not to address a reported problem as part of the intervention. Problems were categorised from the nurses’ notes following the intervention; categories were agreed between the two nurses through discussion.
