# Supplementary figures and images for: The UPBEAT Nurse-Delivered Personalized Care Intervention for People with Coronary Heart Disease Who Report Current Chest Pain and Depression: A Randomised Controlled Pilot Study
Source: PLoS One. 2014 Jun 5;9(6):e98704. doi: 10.1371/journal.pone.0098704 (PMC4047012; doi:10.1371/journal.pone.0098704)

Appendix 3: EQ-5D score and QALY gain


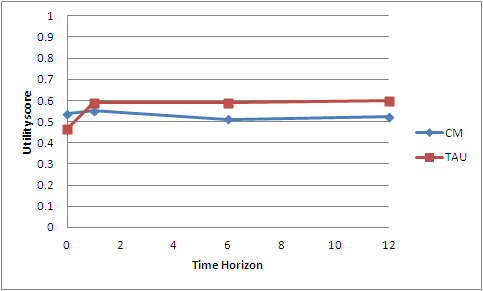

Supplement: Appendix S3 — EQ-5D score and QALY gain. (DOCX) [file pone.0098704.s003.docx]
