# Supplementary material for: The UPBEAT Nurse-Delivered Personalized Care Intervention for People with Coronary Heart Disease Who Report Current Chest Pain and Depression: A Randomised Controlled Pilot Study
Source: PLoS One. 2014 Jun 5;9(6):e98704. doi: 10.1371/journal.pone.0098704 (PMC4047012; doi:10.1371/journal.pone.0098704)
Supplement: Appendix S4 — Service Use & Costs (£) at baseline, 6-month and 12-month follow-ups (by randomisation group). Measured using the Client Service Receipt Inventory (CSRI). (DOCX) [file pone.0098704.s004.docx]

Table 6 Service Use & Costs (£) at baseline, 6-month and 12-month follow-ups (by randomisation group)

| **TYPE OF SERVICE** | **PC** | | | | | | **TAU** | | | | | |  |
| --- | --- | --- | --- | --- | --- | --- | --- | --- | --- | --- | --- | --- | --- |
|  | *Baseline (N=41)* | | *6-month follow-up (N=35)* | | *12-month follow-up (N=32)* | | *Baseline (N=40)* | | *6-month follow-up (N=39)* | | *12-month follow-up (N=37)* | | |
|  | N^1^ (%) | Mean^2^ (SD) | N^1^ (%) | Mean^2^ (SD) | N^1^ (%) | Mean^2^ (SD) | N^1^ (%) | Mean^2^ (SD) | N^1^ (%) | Mean^2^ (SD) | N^1^ (%) | Mean^2^ (SD) | |
|  |  |  |  |  |  |  |  |  |  |  |  |  | |
| A&E | 7(17) | 63(0) | 4(11) | 94(63) | 2(6) | 125(89) | 13(33) | 77(38) | 8(21) | 110(73) | 6(16) | 73(26) | |
| Day hospital | 3(7) | 347(309) | 1(3) | 703(.) | 2(6) | 440(371) | 4(10) | 233(149) | 3(8) | 238(149) | 2(5) | 396(345) | |
| Inpatient care | 11(27) | 1,768(1,609) | 1(3) | 805(.) | 6(19) | 1,118(704) | 14(35) | 6,105(11,201) | 7(18) | 1,418(1,321) | 8(22) | 3,690(4,013) | |
| Outpatient care | 30(73) | 947(2,064) | 21(60) | 253(139) | 19(59) | 664(888) | 30(75) | 1,008(1,765) | 30(77) | 694(735) | 25(68) | 565(389) | |
| GP | 37(90) | 250(518) | 27(77) | 171(165) | 27(84) | 210(383) | 36(90) | 237(288) | 36(92) | 188(233) | 32(86) | 158(218) | |
| Psychiatrist | 0 (0) | - | 0 (0) | - | 0 (0) | - | 0 (0) | - | 1(3) | 656(.) | 0 (0) | - | |
| Other c/b doctor | 0 (0) | - | 0 (0) | - | 0 (0) | - | 1(3) | 100(.) | 0 (0) | - | 0 (0) | - | |
| District nurse | 0 (0) | - | 2(6) | 238(285) | 1(3) | 146(.) | 1(3) | 641(.) | 1(3) | 366(.) | 3(8) | 250(339) | |
| Practice nurse | 25(61) | 15(19) | 13(37) | 14(8) | 16(50) | 14(12) | 23(58) | 25(51) | 17(44) | 21(42) | 18(49) | 11(10) | |
| Mental health nurse | 1(2) | 112(.) | 0 (0) | - | 0 (0) | - | 0 (0) | - | 0 (0) | - | 0 (0) | - | |
| Health visitor | 0 (0) | - | 0 (0) | - | 1(3) | 100(.) | 0 (0) | - | 0 (0) | - | 0 (0) | - | |
| Other nurse | 0 (0) | - | 1(3) | 58(.) | 1(3) |  | 0 (0) | - | 1(3) | 19(.) | 1(3) | 173(.) | |
| Psychologist | 1(2) | 972(.) | 2(6) | 324(0) | 1(3) | 972(.) | 0 (0) | - | 1(3) | 162(.) | 2(5) | 162(115) | |
| Counselor | 2(5) | 569(434) | 2(6) | 88(31) | 0 (0) | - | 5(13) | 210(189) | 4(10) | 334(197) | 2(5) | 212(258) | |
| Occup. therapist | 0 (0) | - | 1(3) | 42(.) | 1(3) | 84(.) | 0 (0) | - | 0 (0) | - | 0 (0) | - | |
| Physiotherapist | 3(7) | 504(436) | 0 (0) | - | 4(13) | 194(214) | 1(3) | 42(.) | 2(5) | 53(15) | 4(11) | 562(970) | |
| Other therapist | 0 (0) | - | 0 (0) | - | 2(6) | 28(8) | 2(5) | 42(38) | 2(5) | 57(48) | 1(3) | 100(.) | |
| Social worker | 0 (0) | - | 0 (0) | - | 0 (0) | - | 0 (0) | - | 1(3) | 71(.) | 1(3) | 107(.) | |
| Housing worker | 0 (0) | - | 1(3) | 26(.) | 0 (0) | - | 0 (0) | - | 0 (0) | - | 0 (0) | - | |
| Home care worker | 2(5) | 227(107) | 2(6) | 1,210(0) | 1(3) | 1,814(.) | 2(5) | 1,361(1069) | 1(3) | 454(.) | 2(5) | 6,237(8,286) | |
| Care attendant | 0 (0) | - | 3(9) | 2,407(3,487) | 1(3) | 605(.) | 0 (0) | - | 0 (0) | - | 1(3) | 1210(.) | |
| Support worker | 0 (0) | - | 0 (0) | - | 0 (0) | - | 1(3) | 103(.) | 0 (0) | - | 1(3) | 182(.) | |
| Voluntary worker | 0 (0) | - | 0 (0) | - | 1(3) | 288(.) | 0 (0) | - | 1(3) | 10(.) | 0 (0) | - | |
| Day centre | 0 (0) | - | 2(6) | 138(172) | 0 (0) | - | 1(3) | 518(.) | 3(8) | 238(219) | 1(3) | 1042(.) | |
| Other c/b service | 4(10) | 563(222) | 0 (0) | - | 1(3) | - | 4(10) | 1,929(2,342) | 0 (0) | - | 0 (0) | - | |
| Personal care | 2(5) | 150(35) | 0 (0) | - | 0 (0) | - | 0 (0) | - | 0 (0) | - | 0 (0) | - | |
| Help in/around home | 13(32) | 337(306) | 7(20) | 332(252) | 8(25) | 428(366) | 8(20) | 259(339) | 4(10) | 388(269) | 7(19) | 150(114) | |
| Help outside home | 8(20) | 233(187) | 2(6) | 63(18) | 4(13) | 50(29) | 5(13) | 75(61) | 3(8) | 75(66) | 3(8) | 100(43) | |
| Other help | 1(2) | 750(.) | 0 (0) | - | 0 (0) | - | 3(8) | 650(677) | 0 (0) | - | 0 (0) | - | |
|  |  |  |  |  |  |  |  |  |  |  |  |  | |
| Total cost |  | 1,773(2,498) |  | 832(1,383) |  | 1,088(1,320) |  | 3,604(7,852) |  | 1,191(1,168) |  | 2,014(3,246) | |
|  |  |  |  |  |  |  |  |  |  |  |  |  | |
| **CM, nurse-led case management; TAU, treatment as usual; c/b, community-based**  **^1^Number of contacts/attendances (rounded to the nearest integer, amongst service users only)**  **^2^Mean costs for the number of attendances/overnight stays for hospital services, mean costs for the number of contacts for community services and mean costs for the number of help hours per week for informal care, in the last 6 months per patient (rounded to the nearest integer , amongst service users only)** | | | | | | | | | | | | | |
