# Supplementary material for: The UPBEAT Nurse-Delivered Personalized Care Intervention for People with Coronary Heart Disease Who Report Current Chest Pain and Depression: A Randomised Controlled Pilot Study
Source: PLoS One. 2014 Jun 5;9(6):e98704. doi: 10.1371/journal.pone.0098704 (PMC4047012; doi:10.1371/journal.pone.0098704)

**Appendix 5** Distribution of the cost-effectiveness point estimates on the cost-effectiveness plane


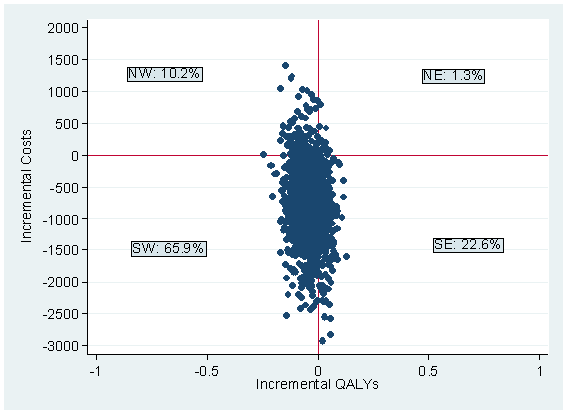

Supplement: Appendix S5 — Distribution of the cost-effectiveness point estimates on the cost-effectiveness plane. (DOCX) [file pone.0098704.s005.docx]
