# Supplementary material for: The UPBEAT Nurse-Delivered Personalized Care Intervention for People with Coronary Heart Disease Who Report Current Chest Pain and Depression: A Randomised Controlled Pilot Study
Source: PLoS One. 2014 Jun 5;9(6):e98704. doi: 10.1371/journal.pone.0098704 (PMC4047012; doi:10.1371/journal.pone.0098704)
Supplement: Protocol S1 — Trial protocol. (DOC) [file pone.0098704.s007.doc]

**A pilot randomised controlled trial (RCT) of case management for depressed primary care patients with symptomatic coronary heart disease**

***Background***

The World Bank and WHO have predicted that coronary heart disease (CHD) and depressive disorder will be the two top causes of global health burden and disability by 2020^1^. Depression is twice as prevalent in CHD compared to the general population ^2^. When CHD and depression co-exist, the conditions interact resulting in worse outcomes; patients with CHD and depression have an approximate two-fold increase in morbidity and mortality ^3-5^, and significantly worse health-related quality of life ^6^. It is therefore important to address the care of patients with both conditions whose burden at an individual level is often immense.

Good mental health care has been a priority area for the NHS since the National Service Framework^7^. The importance of prompt recognition and best management of common mental health problems such as depression has also been stressed in a range of reports from the Department of Health, including “The NHS Plan”, “Treatment choice in Psychological Therapies and Counselling”, “The NSF for mental health- 5 years on”, “Our Health, our care our say: a new direction for community services”, New Horizons and many NICE guidelines^8^ ^-12^.The recent Department of Health guide “Improving Primary Care Mental Health Services” ^13^, provides guidance on best practice and Practice Based Commissioning (PBC) for depression services in long-term conditions such as stroke, CHD and diabetes. The guidance states that depression is associated with a 50% increase in the costs of long term medical care after controlling for the severity of the physical illness^14^. Some of these costs are associated with the effects of depression on adverse health risk behaviours such as smoking, diet and lack of exercise and a lack of compliance with self care regimes. Depression as cause or consequence of physical illness such as CHD may exacerbate the perceived severity of symptoms and distress and increase the utilisation of services^14^. Treating depression as well as helping improve outcomes for depression can also reduce health costs in people with physical illness^14;15^.To date, most of this type of research has been conducted in North America from insurance data, so this type of research is needed in the NHS

Depression increases the incidence of CHD in healthy subjects. Two recent systematic reviews with meta-analyses have shown that depression among healthy individuals increases the risk of subsequent CHD by approximately 60% (Relative Risk (RR) = 1.64), even after controlling for other cardiac risk factors^16;17^. One review found a dose effect of depression with the higher risk associated with clinical depression (i.e. assessed by a clinical procedure, RR= 2.69) compared to depressive symptoms (i.e. measured by standardised scale, RR=1.49) ^16^.

Depression in individuals with established CHD, predicts worse medical outcomes, such as further coronary events (Odds Ratio (OR) = 2.0), death (O.R = 2.6) and greater impairment in health-related quality of life ^2;3^. The precise mechanisms by which depression influences cardiac outcomes are unclear, though both behavioural and physiological pathways have been proposed ^18;19^. There is also recent evidence that individuals who develop depression following acute coronary syndrome (ACS), as opposed to those with depression that pre-dates the ACS, may be at particularly high risk of worse cardiac outcomes^5;15;20^.

Symptomatic Coronary Heart Disease (sCHD) often causes functional limitation and distressing symptoms, is often life threatening and requires long-term management, mostly in primary care. Most patients with sCHD have a documented history of myocardial infarction or coronary artery disease shown at angiography. CHD causes 70% of heart failure. sCHD mainly comprises three groups of patient with; chronic stable angina, post myocardial infarction and heart failure with a hierarchy of physical and emotional effects in all these groups. The prevalence of CHD in Lambeth, Southwark and Lewisham Primary Care Trusts (PCTs) is 3.5%, ascertained from CHD registers in practices kept for the General Medical Services Contract. Concurrent physical illness is known to reduce the likelihood of major depression being recognised by GPs^21^;^22^.

Regarding the management of co-morbid depression and CHD, every patient should be considered as a whole person with physical, psychological and social needs^23^. GPs are now remunerated in the Quality and Outcomes Framework (QoF) in their General Medical Services (GMSII) for screening their CHD patients for depression. They are also remunerated for asking their patients at baseline to complete one of three possible self report psychiatric diagnostic scales to assess the severity of depression (www.nhsalliance.org) to help determine best management. It is currently uncertain how well this will be implemented in everyday general practice. DH has set out additional guidance on assessing and managing long term illness which includes assessing psychosocial factors that may need addressing (ref). It is not clear how GPs and Practice Nurses will best manage the additional depression that will be identified in their CHD patients.

This pilot RCT forms part of a National Institute of Health Research funded Programme of research into the relationship between coronary heart disease and depression. The overall Programme consists of 4 inter-related studies:

1. A 4 year cohort study (in progress)

2. A qualitative study of patients with symptomatic coronary heart disease (sCHD) and distress/ depression (completed)

3. A qualitative study of general practitioners (GPs) and practice nurses (PNs) (completed)

4. A pilot randomised controlled trial (RCT) of case management for depressed primary care patients with sCHD

The intervention for the pilot RCT derives from the findings of the two qualitative studies (2&3 above). Case management has been shown to improve outcomes for depression in primary health-care settings ^24^, but there has been no research to determine whether it is effective in patients with symptomatic coronary heart disease. Case management has been defined as ‘taking responsibility for following-up patients; determining whether patients were continuing the prescribed treatment as intended; assessing whether depressive symptoms were improving; taking action when patients were not adhering to guideline based treatment or were not showing expected improvement’ .^25^ It consists of five essential components ^24^:

1. identification of patients in need of services
2. Assessing individual patient’s needs
3. Developing a treatment plan
4. Coordination of care
5. Monitoring outcomes and altering care when favourable outcomes are not achieved

An RCT is necessary to determine whether case management for this population is more effective than treatment as usual both in terms of depression and cardiac outcomes.

A pilot RCT is needed first to inform the design of the definitive RCT.

***Objectives***

The objectives of this pilot are:

1. *Sample size calculation*

Estimates of the location of the mean and variability around the mean (standard deviation) for the primary outcome measure (see measures) will be calculated. A conservative estimate using the 95% upper confidence limit will be used to inform the sample size calculation of the definitive RCT.

1. *To enable selection of the most appropriate primary and secondary outcome measures*. The pilot will also allow potential secondary outcome measures to be assessed.
2. *Integrity of the study protocol*

The pilot will allow all procedures of the definitive RCT to be piloted. This will include testing:

- inclusion/exclusion criteria
- training of staff in the administration and assessment of the intervention.

1. *Testing data collection forms and questionnaires*

This will ensure that the questionnaires are acceptable to the participants, are comprehensible, appropriate, clearly defined and presented in a consistent manner. Patient information documents and consent forms will also be tested. Inter-rater reliability between researchers will be tested.

1. *Randomization procedure*

The randomisation process and acceptability of randomisation to primary care professionals and participants will be tested.

1. *Recruitment and consent*

The recruitment method will be tested and the consent rate for participants into the study calculated. Barriers to recruitment of both practices and participants will be explored. Follow-up rates will be calculated.

1. *To determine the acceptability of the intervention and the trial to practices and participants.* To determine the possible sources of contamination, and to develop a standardised manual for case management for use in the definitive RCT. To make an informal assessment of the degree to which the intervention can be standardised and whether therapist effects are likely to be a major factor.

***Method***

This will be a pilot randomised controlled trial conducted in primary care. A descriptive account of the process and participants will be recorded.

*Setting and Practice recruitment*

The study will be carried out in primary care. Practices in South London will be recruited via the Greater London Primary Care Research Network (PCRN-GL) and invited to participate in the pilot study. We estimate from the results of the cohort study that we will need 10-15 practices each with around 10,000 patients in order to randomise between 30 and 50 participants per arm.

*Participant recruitment*

All patients on practice case registers for CHD will be asked by their GP for consent to contact from a researcher. Those consenting will be contacted by a researcher and assessed for depression using the Patient Health Questionnaire-2^26^ and for symptoms relating to CHD using the Modified Rose Angina Questionnaire ^27^. Patients scoring 3 or more on the PHQ-2, and with symptomatic CHD will then be assessed further using the Hospital Anxiety and Depression Scale (HADS)^28^. If they score >9 on the depression scale of HADS they will be eligible to participate in the study. Those consenting to participate will then be randomised to either to the intervention (case management) or the treatment as usual (TAU) arm of the study.

We will aim to recruit between 30 and 50 patients in each arm of the study.. This is in line with the minimum of 12 per group suggested by Julious (2005) for pilot studies.

*Inclusion Criteria (Practices)*

· Practice keeps a register of patients with CHD for the Quality and Outcomes Framework (QOF) and is willing to liaise over patients in the case management arm when necessary

*Inclusion Criteria (Participants)*

· Symptomatic CHD as scored on the modified Rose Angina Questionnaire

· A score >9 on the depression part of the Hospital Anxiety and Depression Scale

· Aged 16 years or over

·

*Exclusion Criteria (Participants)*

· Temporary registrations

· Actively suicidal patients

· Psychotic depression as evidenced by delusions and/or hallucinations

Non-English speaking

Participants currently in hospital for treatment of their CHD

Up to 20 group practices of around 10,000 patients each from South London will be recruited by the Greater London Local Research Network (GLLRN) to participate in the study.

*Intervention*

The intervention will be case management delivered by two clinically qualified researchers (a community psychiatric nurse and a practitioner health psychologist who is also a registered nurse)

The case managers will arrange to meet with each participant randomised to the intervention arm for an initial assessment. At the assessment, the case managers will draw-up with the participants a personalised care plan (PCP) (see appendix). This will take a holistic approach to the participant’s current mental, physical and social problems. A copy of the PCP will be kept by the participant, a copy by the case manager and a copy sent to the participant’s GP. The case manager will help the participant choose up to two problems to work on using goal setting techniques to enhance their self- efficacy in self management. The case managers will also provide written information where appropriate about depression and other appropriate local resources for participants to access. The initial assessment is likely to last up to an hour. The case manager will liaise with other health professionals involved in the participant’s care as appropriate.

They will be followed up by the case manager by telephone (or email, if the patient prefers) for 6 months. Initially contact is likely to be at least weekly, but later may be two-weekly. During the follow-up, the participant’s PCP will be reviewed with particular emphasis on mutually prioritised problems and any new goals set in collaboration with the participant as appropriate.

*Control*

Participants randomised to the TAU control group will receive treatment as usual by their GP and any other relevant professionals.

*Measures*

Participants will be followed up at 1-, 6- and 12-months post randomisation. The same measures will be applied at each follow-up

The following measures will be piloted:

| **Outcome Parameter** | **Instruments** |
| --- | --- |
|  |  |
|  |  |
| **Primary Outcome** |  |
| Depression | HADS |
| **Secondary Outcome** |  |
| Depression | PHQ-9 |
| Coronary Heart Disease | Modified Rose Angina Questionnaire, Specific Activity Schedule |
| Quality of Life | Euroqol 5D, Medical Outcomes Survey Short Form-12 (SF-12) |
| Adherence to medication | Adapted version of Morisky adherence questionnaire |
| Life events | List of Threatening Events Questionnaire |
| Social problems | Social Problems Questionnaire |
| Health Service Utilisation | Client Service Receipt Inventory (CSRI) |
| Illness Perceptions | Brief Illness perceptions Questionnaire |
| Participants problem priorities | Psychlops |

In order to evaluate the process of delivering the intervention, focus groups of participants in the intervention arm and primary care professionals will be conducted to explore their experiences, views on the intervention, and their experience of participating in this study.

*Randomisation*

The unit of randomisation will be the participant. Randomisation will be conducted independently by the Clinical Trials Unit at the Institute of Psychiatry.

*Blinding*

Researchers will be blind to randomisation status. Researchers will be asked to give their opinion on randomisation status to determine whether blinding is adequate.

*Statistical analysis*

Descriptive analyses will be used to provide summary estimates of outcome measures, focussing on the dropout rate at each time point, the order of magnitude of the effect of the intervention and the variability of the outcome measure at baseline. While the sample size will not be sufficient to test clustering effects formally, sources of clustering will be identified so that they can be taken into account in the definitive RCT.

*Ethical considerations*

Participants may withdraw from the study at any time and without giving any reason without their care being affected. Any participant thought to be suicidal, either by the researchers (as assessed on depression questionnaires), or by the case managers will have their GP informed immediately. Case managers will have regular supervision by clinicians experienced in general practice and psychiatry employed on the research team. The team also has an experienced consultant cardiologist to provide advice where appropriate.

*Study supervision*

The conduct of the programme will be overseen by a multidisciplinary steering group. Professor Roger Jones, Professor of General Practice, King’s College London will Chair an independent Steering Group. A senior clinical researcher with expertise in running RCTs will coordinate the trial.

.A DMEC is considered necessary if there are at least 2 of the following:

1) the trial is intended to provide definitive information on effectiveness;

2) prior data suggests that intervention could be unacceptably toxic;

3) there are safety implications (eg when outcome is mortality);

4) one would want the trial to stop early if it provided definitive evidence for effectiveness;

(summary of main points from Ellenberg, Fleming and DeMets).

Since none of these apply in this pilot study,iit is proposed that an independent data monitoring and ethics committee (DMEC) will not be employed unless required by the Steering Committee.

Reference List

1. Murray CJ,.Lopez AD. Alternative projections of mortality and disability by cause 1990-2020: Global Burden of Disease Study. *Lancet.349(9064):1498-504,* 1997.

2. Davidson W KDBTCRCRCJCSFEF-SNFKFEGAKWKPKRKHKRLFRSDaSJ. Assessment and treatment of depression in patients with cardiovascular disease: National Heart, Lung, and Blood Institute Working Group report. *Psychosom.Med.* 2004;**68**:645-50.

3. Barth J, Schumacher M, Herrmann-Lingen C. Depression as a risk factor for mortality in patients with coronary heart disease: a meta-analysis. *Psychosomatic Medicine.66(6):802-13,* 2004;-Dec.

4. Nicholson A, Kuper H, Hemingway H. Depression as an aetiologic and prognostic factor in coronary heart disease: a meta-analysis of 6362 events among 146 538 participants in 54 observational studies. *European Heart Journal.27(23):2763-74,* 2006.

5. van Melle JP, de Jonge P, Spijkerman TA, Tijssen JG, Ormel J, van Veldhuisen DJ *et al*. Prognostic association of depression following myocardial infarction with mortality and cardiovascular events: a meta-analysis. *Psychosomatic Medicine.66(6):814-22,* 2004;-Dec.

6. Stafford L, Berk M, Reddy P, Jackson HJ. Comorbid depression and health-related quality of life in patients with coronary artery disease. [Review] [107 refs]. *Journal of Psychosomatic Research.62(4):401-10,* 2007.

7. Department of Health. Modern standards and service models- Mental Health National Service Framework. 1999. London, Department of Health.

Ref Type: Report

8. Department of Health. The NHS Plan: a plan for investment, a plan for reform. 010481829. 2000. Department of Health.

Ref Type: Report

9. Department of Health. Treatment choice in Psychological Therapies and Counselling. 23044. 2001. Department of Health.

Ref Type: Report

10. Appleby L. The national service framework for mental health-five years on. 265907. 2004. Department of Health.

Ref Type: Report

11. Department of Health. Our Health, our care our say: a new direction for community services. 2006. Norwich, HMSO.

Ref Type: Report

12. National Collaborating Centre for Mental Health. Depression: Management of depression in primary and secondary care. 23. 2004. London, National Institute for Clinical Excellence.

Ref Type: Report

13. Raistrick H and Richards D. Designing Primary Care Mental Health Services. 2006. Department of Health.

Ref Type: Report

14. Katon W, VON KORFF M, Lin E, Simon G, Ludman E, Bush T *et al*. Improving primary care treatment of depression among patients with diabetes mellitus: the design of the pathways study. *General Hospital Psychiatry.25(3):158-68,* 2003;-Jun.

15. Dickens C, McGowan L, Percival C, Tomenson B, Cotter L, Heagerty A *et al*. New onset depression following myocardial infarction predicts cardiac mortality. *Psychosomatic Medicine.70(4):450-5,* 2008.

16. Bultmann U, Rugulies R, Lund T, Christensen KB, Labriola M, Burr H. Depressive symptoms and the risk of long-term sickness absence: a prospective study among 4747 employees in Denmark. *Social Psychiatry & Psychiatric Epidemiology.41(11):875-80,* 2006.

17. Wulsin LR,.Singal BM. Do depressive symptoms increase the risk for the onset of coronary disease? A systematic quantitative review. [Review] [53 refs]. *Psychosomatic Medicine.65(2):201-10,* 2003;-Apr.

18. Carney RM, Blumenthal JA, Freedland KE, Youngblood M, Veith RC, Burg MM *et al*. Depression and late mortality after myocardial infarction in the Enhancing Recovery in Coronary Heart Disease (ENRICHD) study. *Psychosomatic Medicine.66(4):466-74,* 2004;-Aug.

19. Rozanski A, Blumenthal JA, Kaplan J. Impact of psychological factors on the pathogenesis of cardiovascular disease and implications for therapy. [Review] [279 refs]. *Circulation.99(16):2192-217,* 1999.

20. Grace SL, Abbey SE, Kapral MK, Fang J, Nolan RP, Stewart DE. Effect of depression on five-year mortality after an acute coronary syndrome. *American Journal of Cardiology.96(9):1179-85,* 2005.

21. Freeling P, Rao BM, Paykel ES, Sireling LI, Burton RH. Unrecognised depression in general practice. *British Medical Journal Clinical Research Ed.* 1985;**290**:1880-3.

22. Tylee AT, Freeling P, Kerry S. Why do general practitioners recognise major depression in one woman patient yet miss it in another? *Br.J.Gen.Pract.* 1993;**43**:327-30.

23. The Leeuwenhorst Group. The work of the general practitioner. Statement by a working party appointed by the 2nd European Conference on the teaching of general practitioners. *J.R.Coll.Gen.Pract.* 1977;**27**:117.

24. Gensichen J, Beyer M, Muth C, Gerlach FM, VON KORFF M, Ormel J. Case management to improve major depression in primary health care: a systematic review. [Review] [57 refs]. *Psychological Medicine.36(1):7-14,* 2006.

25. Von Korff Msi,.Goldberg Dpe. Improving outcomes in depression : The whole process of care needs to be enhanced. [Editorial]. *BMJ* 2001;**323**:948-9.

26. Kroenke K, Spitzer RL, Williams JB. The Patient Health Questionnaire-2: validity of a two-item depression screener. *Med.Care* 2003;**41**:1284-92.

27. Wu EB, Smeeton N, Chambers JB. A chest pain score for stratifying the risk of coronary artery disease in patients having day case coronary angiography. *International Journal of Cardiology.78(3):257-64,* 2001.

28. Zigmond AS and Snaith RP. The hospital anxiety and depression rating scale. Acta Psychiatrica Scandinavica 67, 361-370. 1983.

Ref Type: Generic
